# Antiviral Activity of Gold/Copper Sulfide Core/Shell Nanoparticles against Human Norovirus Virus-Like Particles

**DOI:** 10.1371/journal.pone.0141050

**Published:** 2015-10-16

**Authors:** Jessica Jenkins Broglie, Brittny Alston, Chang Yang, Lun Ma, Audrey F. Adcock, Wei Chen, Liju Yang

**Affiliations:** 1 Biomanufacturing Research Institute and Technology Enterprise (BRITE), Department of Pharmaceutical Sciences, North Carolina Central University, Durham, North Carolina, United States of America; 2 Department of Physics, University of Texas at Arlington, Arlington, Texas, United States of America; Sun Yat-sen University, CHINA

## Abstract

Human norovirus is a leading cause of acute gastroenteritis worldwide in a plethora of residential and commercial settings, including restaurants, schools, and hospitals. Methods for easily detecting the virus and for treating and preventing infection are critical to stopping norovirus outbreaks, and inactivation via nanoparticles (NPs) is a more universal and attractive alternative to other physical and chemical approaches. Using norovirus GI.1 (Norwalk) virus-like particles (VLPs) as a model viral system, this study characterized the antiviral activity of Au/CuS core/shell nanoparticles (NPs) against GI.1 VLPs for the rapid inactivation of HuNoV. Inactivation of VLPs (GI.1) by Au/CuS NPs evaluated using an absorbance-based ELISA indicated that treatment with 0.083 μM NPs for 10 min inactivated ~50% VLPs in a 0.37 μg/ml VLP solution and 0.83 μM NPs for 10 min completely inactivated the VLPs. Increasing nanoparticle concentration and/or VLP-NP contact time significantly increased the virucidal efficacy of Au/CuS NPs. Changes to the VLP particle morphology, size, and capsid protein were characterized using dynamic light scattering, transmission electron microscopy, and Western blot analysis. The strategy reported here provides the first reported proof-of-concept Au/CuS NPs-based virucide for rapidly inactivating human norovirus.

## Introduction

Human norovirus (HuNoV) is a leading food and waterborne pathogen that causes nonbacterial, acute gastroenteritis outbreaks worldwide [1–3], accounting for more than 21 million illnesses, and contributing to about 70,000 hospitalizations and at least 570 deaths in the United States each year (Centers for Disease Control and Prevention, 2013). Noroviruses are single-stranded RNA, non-enveloped viruses in the *Calicivirdae* family. They are classified into five genogroups (GI to GV) and further subclassified into genotypes and genetic clusters based on their capsid sequence [1]. Their genetic diversity, low (18 particles or less) infectious dose [4], myriad of foodborne and waterborne transmission routes, and prolonged (few hours to several weeks) survival on multiple environmental surfaces [5, 6] lead to frequent epidemics in a variety of residential and commercial settings, including restaurants, schools, nursing homes, and cruise ships [7–12]. In addition, the lack of general population immunity [3, 13]–symptomatic, asymptomatic, and healthy individuals are all capable of spreading HuNoV [14]–greatly increases the likelihood of widespread infection, especially among young children, the elderly, and immunocompromised persons [15, 16].

To effectively prevent norovirus outbreaks, scientists have been working to develop methods for easily detecting the virus and for treating and preventing norovirus infection. However, two of the major challenges in norovirus research are the inability to grow the virus in a cell culture system and the lack of a good animal model system for studying details of how viruses cause illness and testing antiviral agents. In recent years, various viral inactivation strategies have been proposed and studied using norovirus virus-like particles (VLPs) [17] or norovirus surrogates such as murine norovirus, feline calicivirus, and poliovirus [18, 19].

Norovirus VLPs are replication-incompetent, macromolecular protein assemblies with capsid structures and antigenic properties resembling those of native noroviruses [20–22]. Each VLP is ~38 nm in diameter and has repeating arch-like surface features. These arches are formed by 90 dimers of a single capsid protein and contain both a shell and protruding (P) domain. The former houses the capsid’s N-terminus, consisting of 225 residues of the 530 amino acid (aa) sequence [23], while the latter forms the topmost P2 domain of the arch-like structure and the P1 domain which connects the shell and P2 domain. The P1 and P2 domains are consisted of the C-terminus and the central regions of the amino acid sequence, respectively [24]. The norovirus VLPs are morphologically and antigenically similar to native virus, and have been widely used in experimental approaches for characterizing these viruses and studying inactivation methods for noroviruses over the past 20 years.

Many of the inactivation strategies strive to prevent intact HuNoV capsids from recognizing their binding sites and entering into host cells to replicate [25] by damaging genomic RNA or capsid proteins [25, 26]. Reported inactivation methods included chemical disinfection [27–30], chitosan additives [31, 32], high pressure homogenization [29], pulsed light [25], UV irradiation [33], variations in pH [34, 35], high temperature treatment [36–39], high pressure treatment [17, 40–42], and radiation treatment [18]. Many of these studies used surrogate viruses in their studies, meanwhile there are an increased number of studies using VLPs in laboratory research for initiative studies of novel/improved methods for inactivation of novoviruses [17, 18], due to their similarity to native virus and availability to laboratory users.

Research has shown that the most promising virucides are those that directly bind to the viral capsid [43], making inactivation via nanoparticles (NPs) a more universal and attractive alternative to other physical and chemical strategies. Metallic NPs owe their broad-spectrum antimicrobial activity to their small size and high surface to volume ratio [44], and many NP types, including silver [45, 46], copper iodide [46], and titanium dioxide [47] can inactivate bacterial cells and norovirus surrogates by interacting with the cell membrane, causing leakage of intracellular substances and cell death [48] or by interacting with (and denaturizing) capsid proteins.

Using norovirus GI.1 (Norwalk) virus-like particles (VLPs) as a model viral system, this study characterized the intrinsic virucidal properties of gold/copper sulfide (Au/CuS) core/shell nanoparticles against GI.1 VLPs for the rapid inactivation of HuNoV. Au/CuS NPs exhibited antimicrobial activity against *Bacillus anthracis* cells [49], but their efficacy as an antiviral has yet to be evaluated. Au/CuS NPs offer many advantages to other metallic NPs, namely lower cost and ready availability compared to silver [48], and to other conventional methods via continuous release of copper ions into solution without reducing agents [46]. In this study, inactivation of VLPs (GI.1) by Au/CuS NPs was evaluated using an absorbance-based ELISA. Both nanoparticle concentration and VLP-NP contact time were investigated as possible variables that affect the virucidal efficacy of Au/CuS NPs. Changes to the VLP particle morphology, size, and capsid protein were characterized using dynamic light scattering, transmission electron microscopy, and Western blot analysis. The strategy reported here provides the first reported proof-of-concept Au/CuS NPs-based inactivation approach for inactivating human norovirus.

## Materials and Methods

### VLPs, Antibodies, and Chemicals

Stock solutions of GI.1 VLPs and monoclonal anti-GI.1 VLP antibody (mAb 3901) at 3.7 and 2.2 mg/mL, respectively, were obtained from Dr. Robert Atmar’s laboratory at the Baylor College of Medicine (Houston, TX). Goat anti-mouse IgG H&L antibody conjugated to horseradish peroxidase (HRP) (0.5 mg/mL) was purchased from Abcam (Cambridge, MA). Goat anti-mouse antibody labeled with IRDye^®^ 800CW infrared dye (0.5 mg) was purchased from LI-COR Biosciences (Lincoln, NE). Phosphate buffered saline (PBS), pH 7.4, was prepared in-house from a 1X (0.01 M) PBS recipe (Cold Spring Harbor Protocols) using NaCl, KCl, Na_2_HPO_4_, and KH_2_PO_4_, all purchased from Fisher Scientific.

### Au/CuS Core/Shell Nanoparticles

Au/CuS core/shell NPs were acquired from Professor Wei Chen’s laboratory at the University of Texas at Arlington. Detailed reaction conditions and procedures have been reported previously [50], but, briefly, the NPs were synthesized using a two-step method by first growing gold NPs as cores using the seeded growth method and then coating these cores with CuS nanoshell. The core/shell structure was confirmed by high resolution transmission electron microscope (HRTEM) imaging of the Au and CuS lattice planes in the core and the shell, respectively, and by spectrophotometric observation of the characteristic absorption peaks for Au and CuS at 531 and 981 nm, respectively [50]. The synthesized Au/CuS NPs were 2–5 nm in diameter with an initial concentration of 83 μM.

### Au/CuS NPs Treatment to GI.1 VLPs

For treatment with NPs, aliquots (17.5 μL) of purified GI.1 VLP suspensions at various concentrations (to reach the final VLP concentrations at 0.37, 3.7, and 5.6 μg/mL) were mixed with 17.5 μL of various Au/CuS NP concentrations ranging from 0.083 to 20.75 μM (to reach the final concentrations ranging from 0.0083 to 2.075 μM) in 1.5 mL microcentrifuge tubes. All solutions were brought to 175.0 μL using 0.01M PBS and continuously agitated at 30–32 RPM using an end-over-end rotator (Dynal Biotech, Inc.; Lake Success, NY) for various treatment times ranging from 10 min to 4 h. Untreated (no NPs) solutions were prepared identically to the VLPs + NPs solutions and used as controls for each treatment time. After treatment, all solutions were centrifuged at 7500 RPM (5283xg) for 5 min using an Eppendorf microcentrifuge (Hamburg, Germany) to separate the NPs and VLPs.

### Evaluation of NPs’ Antiviral Activity

The virucidal efficacy of the Au/CuS NPs was evaluated using an ELISA method previously reported by our group [51]. Briefly, 50.0 μL of untreated or treated VLP solution was dispersed into the wells of a medium-binding 96-well polystyrene plate (Costar^™^ 3591; Corning Incorporated, Corning, NY) and incubated at room temperature for 1 h. 50.0 μL of 0.01M PBS was used as a blank. Each well was washed with 0.01 M PBS and blocked with 100.0 μL of SuperBlock T20 (PBS) Blocking Buffer (Thermo Scientific). The wells were washed with PBS and sequentially treated with 0.2 μg/mL mAb 3901 anti-GI.1 VLP and 0.1 μg/mL HRP-labeled goat anti-mouse IgG antibody solutions for 1 h. The plate was washed twice with 100.0 μL aliquots of 0.01 M PBS + 0.05% Tween^™^ 20 between steps. Following the final washing step, each well was reacted with 100 μL of TMB (3,3’,5,5’-tetramethylbenzidine) Peroxidase Substrate Microwell Substrate System (KPL, Gaithersburg, MD) for 10 min, filled with 50.0 μL of Stop Solution (KPL, Gaithersburg, MD), and the absorbance of each well was read at 450 nm using a SpectraMax M5 plate reader (Molecular Devices, Sunnyvale, CA). Reduced absorbance in NP-treated samples (compared to an untreated control) indicated reduced concentration of intact VLPs due to damage to the capsid surface proteins and associated VLP inactivation.

### Evaluation of VLP Capsid Protein Degradation using Western blot

The effect of NP treatment on the VLP capsid proteins was assessed via immunoblotting. Each NP-treated or untreated VLP solution (5.0 μL containing 0, 0.083, 0.83, and 1.66 μM final concentrations of Au/CuS NPs and 3.7 μg/mL GI.1 VLPs) was mixed with 2.5 μL of 4X LDS Non-Reducing Sample Buffer (Thermo Scientific), 1.0 μL of 1M DTT (Fisher Scientific), and 1.5 μL of DI H_2_O. The denatured samples were resolved onto a precast 1.0mm x 10-well NuPAGE^®^ 4–12% Bis-Tris gel (Life Technologies; Grand Island, NY) using 1X MOPS SDS Running Buffer (Invitrogen; Carlsbad, CA) and transferred to an Odyssey^®^ nitrocellulose membrane (LI-COR Biosciences) using 1X NuPAGE^®^ Transfer Buffer + 10% MeOH. The membrane was blocked with 10.0 mL of 1:1 Blocking Buffer for Fluorescent Western Blotting (Rockland Immunochemicals Inc.; Limerick, PA) + 0.1% PBST. The latter was prepared from HyClone^™^ Dulbecco’s Phosphate Buffered Saline Solution (Fisher Scientific) and Tween^™^ 20. The blocked membrane was sequentially probed with 0.2 μg/mL mAb 3901 anti-GI.1 VLP and 0.07 μg/mL goat anti-mouse IRDye^®^ 800CW antibodies, both diluted in 10.0 mL of 1:1 Rockland Blocking Buffer + 0.1% PBST, and the bound antibodies were visualized with a LI-COR Odyssey Infrared Imaging System. All samples were compared against SeeBlue^®^ Plus2 Prestained Standard (Invitrogen).

### Characterization of Particle Interactions During Treatment

Capsid size was characterized using DLS to determine how the Au/CuS nanoparticles interacted with the VLPs during treatment. 100.0 μL of each NP-treated and untreated VLP solution was analyzed using a Zetasizer Nano ZSP (Malvern Instruments, Westborough, Massachusetts) and disposable BRAND^®^ microcuvettes (Sigma-Aldrich). Suspensions were measured six times each using 10–15 readings per measurement, and the resulting data were averaged to obtain a mean size distribution profile for each solution. Comparing the averaged intensity of the scattered light allows for efficient comparison of VLP dissociation under different conditions [52].

### Transmission electron microscopic (TEM) imaging

TEM were acquired using a FEI Technai G^2^ Twin TEM (Hillsboro, OR) at the Shared Materials Instrumentation Facility (SMIF) at Duke University. To prepare VLPs samples for TEM, a drop of NP-treated or untreated VLP solution was placed on formvar/carbon TEM grids (Electron Microscopy Sciences; Hatfield, PA) for 10 min. All grids were wicked (to remove excess fluid) using filter paper and then stained with 2% uranyl acetate prior to imaging.

### Statistical analysis

Statistical analysis was performed using the general linear model (GLM) procedure of the SAS System 9.2 (SAS Institute Inc., Cary, NC, USA). P < 0.05 was considered as significant different.

## Results and Discussion

### Inactivation Effect of Au/CuS NP Treatment on VLPs

**Fig 1:**
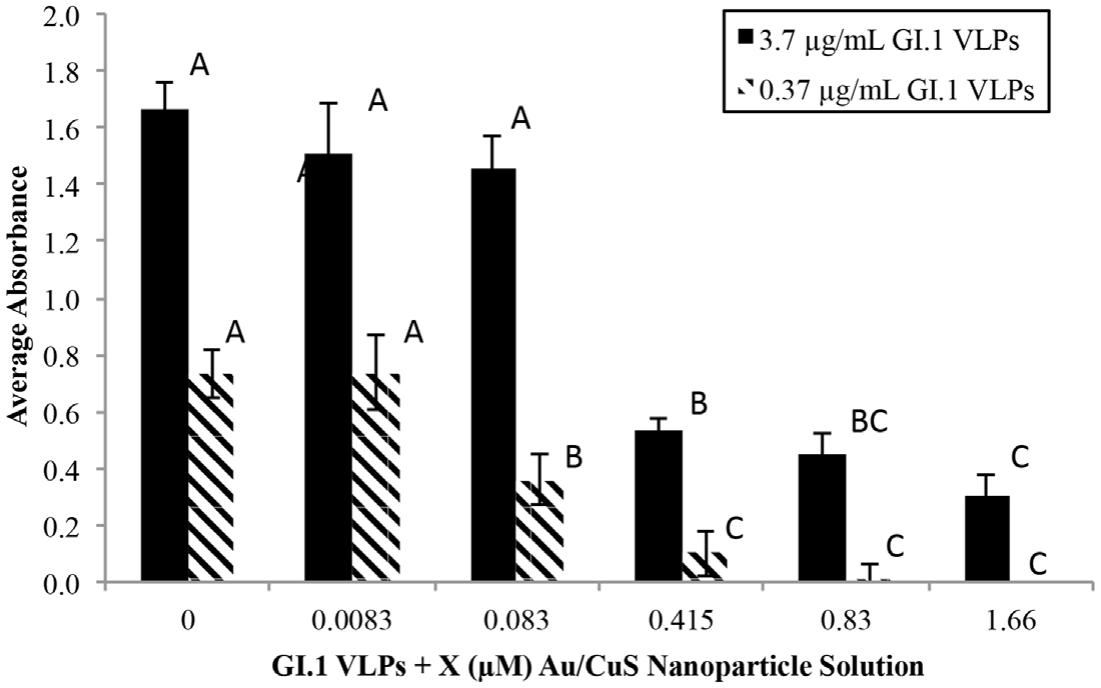
Effect of Au/CuS NP concentration on VLP solution absorbance. Absorbance was measured using the three-hour ELISA, and all solutions contained an equal volume of NPs. Reduced absorbance indicates structural damage to the capsid surface proteins and associated VLP inactivation. In each series, the same letters on the columns indicate no statistically difference, and the different letters indicate statistically different.

We first examined the effect of NPs treatment on VLPs using the ELISA method in which the binding capacity of VLPs to the monoclonal anti-GI.1 VLP antibody (mAb 3901) was evaluated. Fig 1 shows the absorbance signal reductions after VLPs (at two concentration levels: 0.37 and 3.7 μg/mL) were treated with Au/CuS NPs at final concentrations ranging from 0.0083 μM to 1.66 μM for 10 min. For the 0.37 μg/mL VLPs, compared to the untreated control, the inactivation effect was apparent at the treatment with 0.083 μM Au/CuS NPs. Also, there was a marked increase in antiviral activity between 0.083 μM and 0.415 μM, and the VLPs appeared to be completely inactivated at treatments with 0.83 μM and higher NPs. For the denser VLPs at 3.7 μg/mL, treatment with higher concentration of NPs (at 0.415 μM) exhibited a marked antiviral activity, but there were reportable absorbance values at the treatments across the entire tested Au/CuS NP concentration range. This indicates that only partial inactivation was achieved even at high Au/CuS NPs concentrations (up to 1.66 μM).

**Fig 2:**
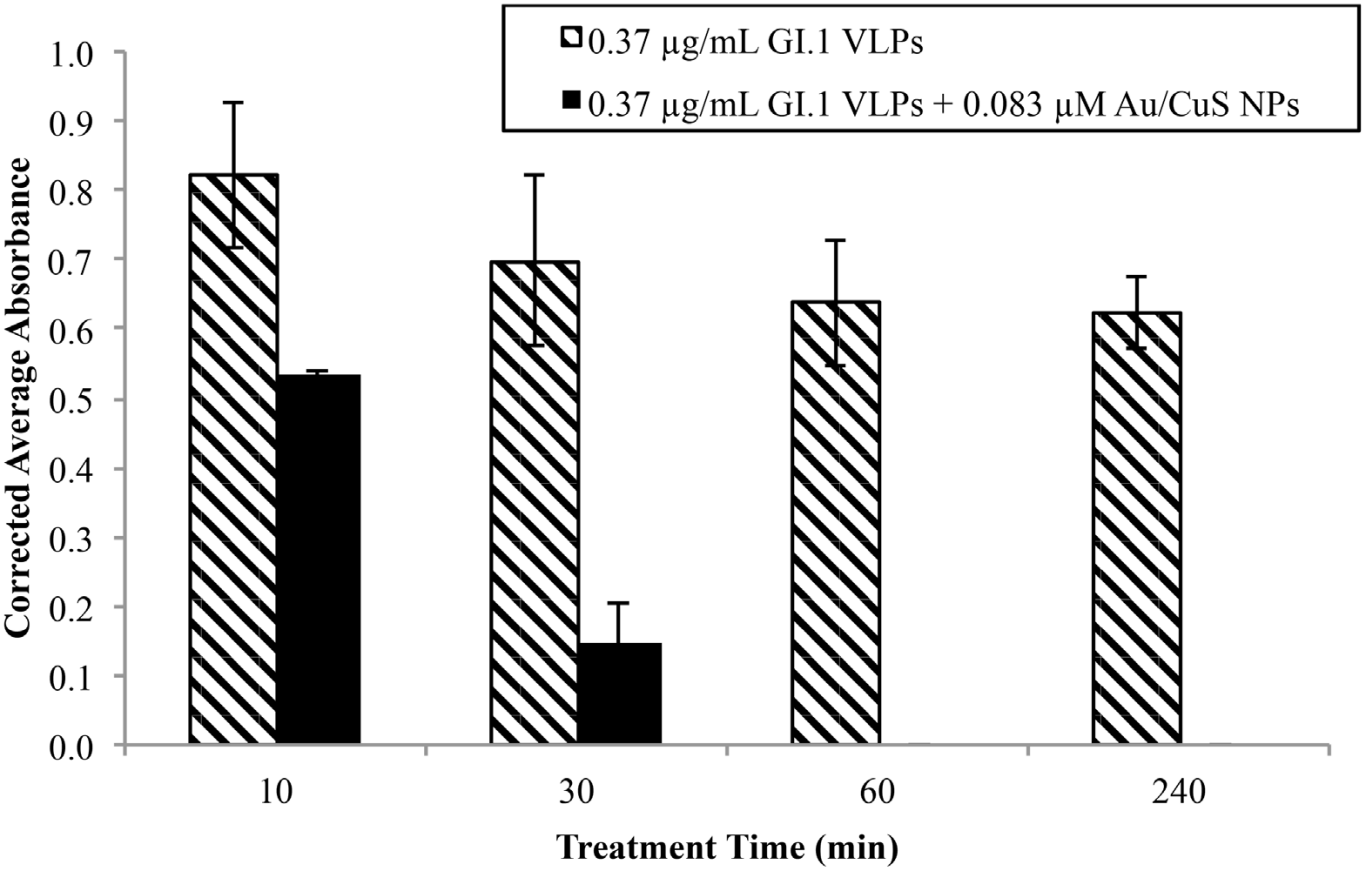
Effect of treatment time on absorbance for VLP solutions treated with 0.83 μM Au/CuS NP. Absorbance was measured using the three-hour ELISA, and new VLP and NP solutions were prepared for each time point. Reduced absorbance suggests structural damage to the capsid surface proteins and is indicative of VLP inactivation.

It turned out that the treatment time had a strong effect on VLP inactivation. Fig 2 shows the absorbance value reductions in VLPs detection by ELISA upon treatment with 0.083 μM Au/CuS for various time periods ranging form 10 min to 4 h. After only 10 and 30 min of treatment, the average absorbance of the treated VLP solutions changes by 35% and 79%, respectively, when compared to the corresponding control solutions, indicating the inactivation of VLPs by Au/CuS NPs was effective and rapid. Also, no absorbance values can be detected for the longer treatment times, while the untreated solutions have similar absorbance values, regardless of treatment time.

The results suggested the NP treated VLPs were morphologically and/or antigenically different from the untreated VLP capsids in that the binding capacity to the detection antibody (mAb 3901) was reduced remarkably. The reduced binding capacity could be due to two possible reasons: NPs might bind to the VLP capsids that may serve to block the binding of mAb3901 to VLPs, or the binding of NPs to VLPs may further cause protein degradation thus the loss/damage of epitopes for mAb 3901 binding.

### Effect of NP Treatment on VLP Capsid Protein

**Fig 3:**
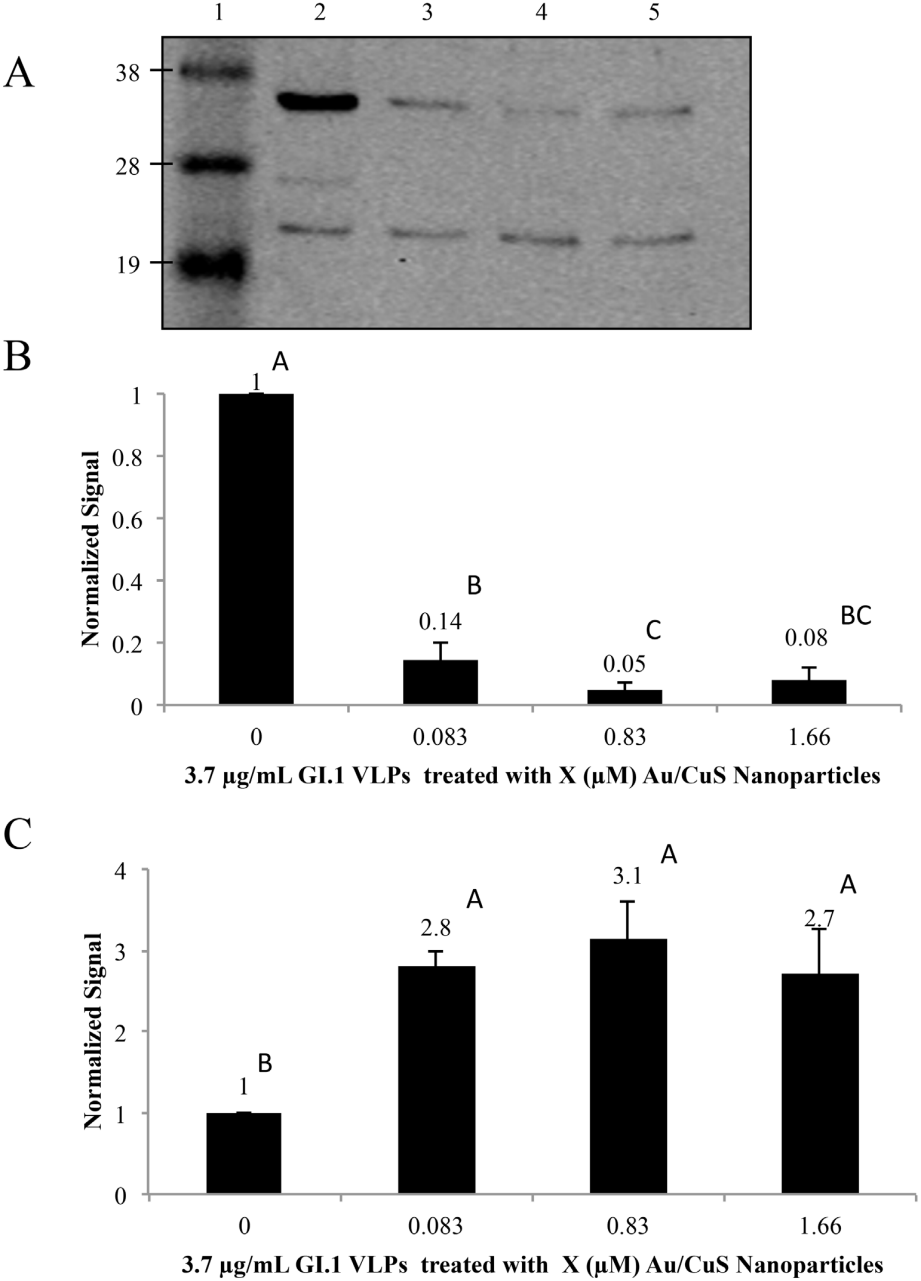
Analysis of capsid surface damage. (A) Immunoblotting with normalized signals (relative to lane 4) for lane 1: MW marker; lane 2: untreated VLP solution; lane 3: VLPs treated with 0.083 μM Au/CuS NPs; lane 4: VLPs treated with 0.83 μM Au/CuS NPs; and lane 5: VLPs treated with 1.66 μM Au/CuS NPs. (B) The relative intensities of the band at approximately 32K. (C) the relative intensities of the band of a small fragment of the capsid protein at approximately 22K. All solutions contain an equal volume of 3.7 μg/mL GI.1 VLPs and/or nanoparticles, and all solutions were centrifuged for 5 min. The same letters on the columns indicate no statistical difference, and the different letters indicate a statistical difference.

We then performed Western blotting to examine protein degradation in VLPs upon Au/CuS NP treatment. Fig 3A shows the immunoblot for VLPs treated with different concentrations of Au/CuS NPs, along with controls. Total proteins were separated by SDS-PAGE and subjected to Western blotting using mAb 3901 primary and fluorescent secondary antibodies. It is known that mAb 3901 can bind to either the full-length (58K) capsid protein or a 32K protein fragment in the P domain [24, 53]. It recognizes a continuous epitope on the C-terminal of the capsid protein, as it was able to bind to the 58K capsid protein and the 32K protein product in Western blot even when the proteins were denatured by boiling prior to analysis [53]. The Western blot here shows that all samples presented a band near 32K, but the relative intensities of the bands obviously varied for the samples treated with different concentrations of NPs. Taking the band intensity of the VLP samples without NP treatment as a base value (taken as 1), the normalized band intensities of the samples treated with different concentrations of NPs are shown in Fig 3B. The results clearly showed that the amount of this 32K protein product reduced significantly as the VLPs were treated with NPs. The treatment with the lowest concentration of NPs (0.83 μM) in the test caused ~86% reduction of this protein, and as the NPs concentration increased to 10 to 20 times higher, it reduced even more and reached a maximum 92–95% reduction. The reduced amount of this protein after NPs treatments suggested that Au/CuS NPs treatment most likely cause degradation (break-down) of this 32K P domain protein.

It is also known that mAb 3901 also recognizes a domain between amino acid 453 and amino acid 495, and the lower band (~22K band) in the Western blot is likely a fragment that contains this sequence. As shown in Fig 3C, this band was less pronounced in the control samples, but was significantly increased in the NPs-treated VLP samples. In fact, normalizing the band intensities relative to the untreated control samples showed that this protein fragment actually increased 2.7- to 3.1-fold in the NPs-treated samples. Again this observation suggested it is likely that the NP treatments caused the 32K P domain protein to break down into smaller fragments. The ~22K product was possibly one of the fragments and contained the domain (aa 453–495) that was recognized by mAb3901. It is not surprising that NPs treatment caused VLP capsid protein degradation, as several other inactivation methods treatments have been reported to cause VLP capsid protein degradation, such as Gamma radiation [18], high pressure treatment [17]. And especially, capsid protein degradation in human norovirus was observed upon contact with copper alloy surface [54] which is closely relevant to this study. At this stage, however, it is unclear about the detailed mechanisms on how Au/CuS NPs can cause the capsid protein to break down, and further studies are necessary to elucidate the detailed mechanisms.

### NPs Treatment Damages Virus Particles

**Fig 4:**
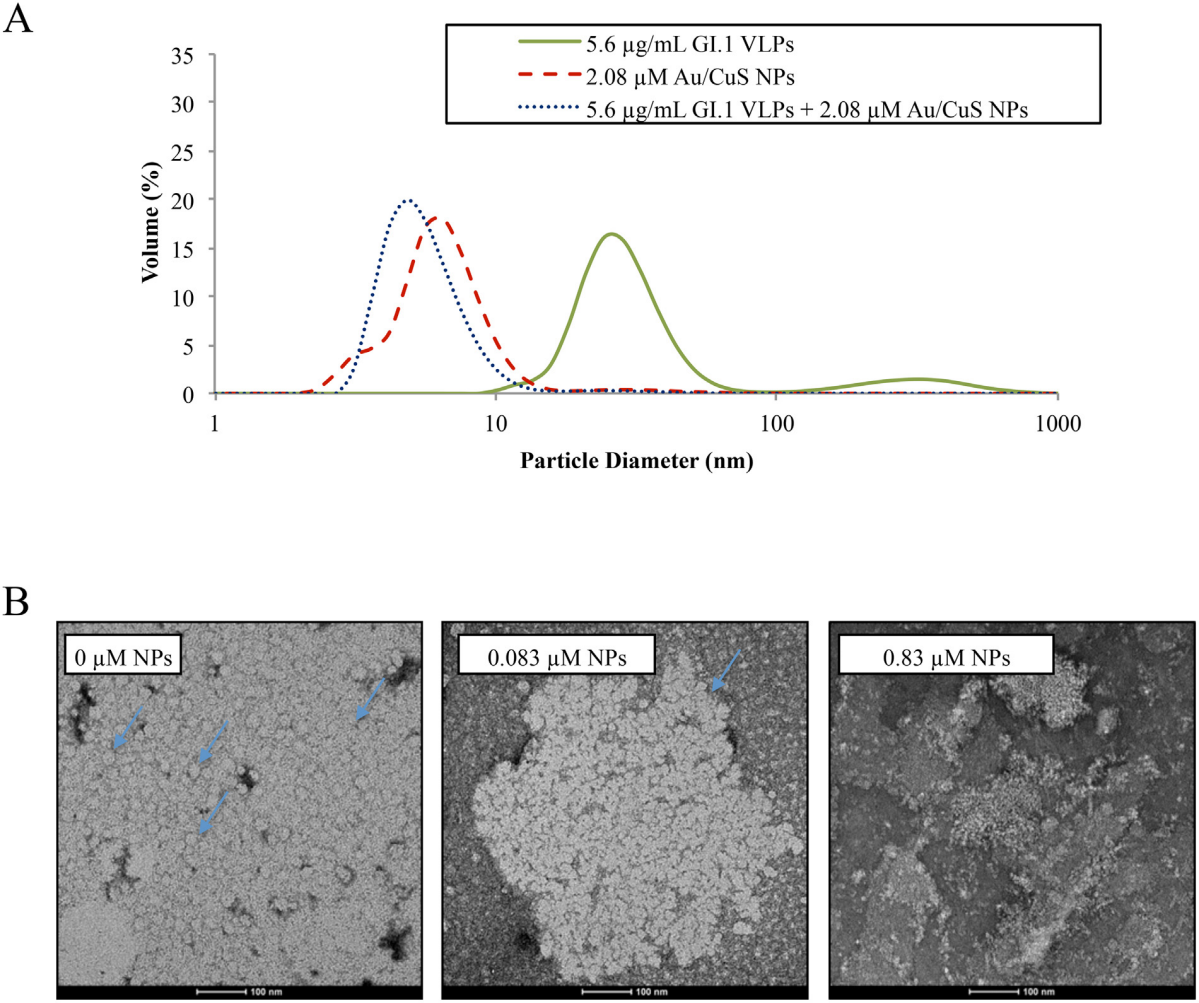
Particle size and appearance after treatment. (A) Mean size of small particles (<10 nm) in treated and untreated VLP solutions. VLP solutions at 5.6 μg/mL were dosed with 2.08 μM Au/CuS NPs, and all solutions were rotated end-over-end and centrifuged for 10 and 5 min, respectively. Particle size was measured using dynamic light scattering. (B) TEM images of untreated VLPs, and VLPs treated with 0.083 μM and 0.83 μM for 30 min. Arrows indicate examples of untreated VLPs which are round shaped circles in light color and aggregated together; Treatment with 0.083 μM NPs caused VLPs lost integrity in the aggregate; Treatement with 0.83 μM NPs caused VLPs to break down into fragments and no intact VLP can be seen.

We further investigated the particle size profiles in VLP suspensions before and post-NPs treatment using dynamic light scattering (DLS). Fig 4 shows the mean particle diameter profiles of VLP suspension, Au/CuS NPs suspension, and the VLP suspension after NP treatment. As expected, for the VLP suspensions, the peak between 10 to 100 nm represented the VLP particles in the suspension, as it is known that the diameter of an assembled norovirus VLP is ~38 nm [53], but smaller and larger particles with 20 to 90 nm diameter were also observed [55]. For the Au/CuS NP suspensions, the peak between 1 and 10 nm indicated the profile of the NPs diameters, which was very close to the previously reported size of 2–5 nm (as determined by SEM/TEM). For VLPs + Au/CuS NPs suspensions, it is obvious that the VLP peak disappeared after 10 min of mixing, suggesting that the intact VLPs may have broken into smaller fragments. Tests on VLP suspensions treated with NPs at other concentrations (0.83 μM and 1.245 μM) showed similar results where the peak between 10 and 100 nm before treatment shifted to <10 nm after NPs treatments (S1 Fig).

Damage to VLPs upon Au/CuS NP treatment was confirmed using TEM (Fig 4B). As shown in the images, the untreated VLPs were intact and readily identifiable with well-defined, regular round shape. However, these features were less distinct after treatment with 0.083 μM Au/CuS NPs. The treatment with the 0.83 μM NPs appeared to break the VLPs’ capsids into fragments and no intact VLPs were observed. The imaging results confirmed that the VLP capsid underwent physical degradation and eventual rupture as the nanoparticle concentration increased from 0.083 μM to 0.83 μM (Fig 4B). This cumulative loss of capsid structural integrity is consistent with the particle size profile changes observed in the DLS spectra.

### Possible Mechanisms of Au/CuS NP Inactivating VLPs

Based on the reported mechanisms of how NPs inactivate bacteria, direct contact and damage to the cell membrane is a common mechanism for many different NPs [56–58]. Other possible routes include suppression of energy metabolism [59], inhibition of enzyme activity and induced oxidative stress [57], increased membrane permeability [60–62], and physical piercing [63]. Although these mechanisms might not all be applicable to how Au/CuS NPs inactivate VLPs, it is likely that some of these mechanisms are involved. The above observations suggested that the Au/CuS NPs may inactivate the VLPs by direct contact/binding to the VLPs and further physically damaging the capsid.

Considering the core/shell structure of the Au/CuS nanoparticles, the CuS shell is the component that most likely interacts with the VLP capsid. Little is known about the antimicrobial activity of pure CuS nanoparticles based on published literature. However, another member of the Cu compound family, the CuO NP, has recently been reported to show antimicrobial activity to several types of microorganisms [64–66]. Based on the action of CuO NPs and other metal oxide NPs (such as ZnO), the release of soluble metal ions (such as Cu^2+^, Zn^2+^) from the NPs largely influenced their toxicity [64]. Our previous study on Au/CuS NPs inactivation of *B*. *anthracis* spores and cells showed that Au/CuS NPs bind to and damage the cell membrane, creating an osmotic imbalance, efflux of cytoplasmic content, and associated cell rupture and death [49]. More relevant studies using copper alloys as antiviral surfaces for murine norovirus—an HuNoV surrogate—showed the virus was destroyed within 60 min [67], with the largest difference in inactivation rate occurring within the first 30 min of contact [68]. These studies reported that the mechanism of copper inactivation of noroviruses involves both degradation of the RNA and destruction of the capsid. A more recent study also reported that exposure to copper alloys destructed the capsid and genome of GII.4 human norovirus [54]. These reports suggested that the Cu component may be a key factor controlling the antiviral activity of Au/CuS NPs to VLPs. And our observations on VLPs’ capsid protein degradation and the loss of capsid integrity by Au/CuS NPs treatment are consistent with the observations reported by these relevant studies [54, 67–69].

It is also possible that an intact NP can diffuse across the cell membrane or virus capsid, or that Cu^2+^ solute from the NPs can enter cells through the transport and ion/voltage-gated channels [70]. While NPs themselves can interact with oxidative organelles or redox active proteins to induce reactive oxygen species (ROS) in cells, Cu^2+^ produced by the NPs can also induce ROS by various chemical reactions, and ROS can break DNA strands and alter gene expression [64]. Another possible mechanism is that Cu^2+^ can chelate with biomolecules or dislodge the metal ions in some metalloproteins, leading to dysfunctional proteins and further cell inactivation [64]. These mechanisms are likely applicable to how Au/CuS NPs interact with VLPs, leading to capsid protein degradation and breakdown. However, as the study of Au/CuS NPs’ antiviral activity is in a very early stage, its detailed mechanisms are not fully understood. Further studies using different viruses and experimental conditions, systematic comparison with pure Au NPs, CuS NPs, and other similar metal NPs, are necessary to fully understand the virucidal properties of Au/CuS NPs.

This study only demonstrated the proof-of-concept that Au/CuS NPs exhibited antiviral activity to norovirus VLPs, and much more work is still needed to apply the concept to a practical approach. Nevertheless, the observations here present both challenges and opportunities for further investigations. The challenges would be to elucidate the detailed mechanisms about Au/CuS NPs’ antiviral activity to VLPs, and to investigate its antiviral activity to norovirus surrogates and native human noroviruses. Since using VLPs as the test model would not be possible to study the effect of Au/CuS NPs on the infectivity of noroviruses, further studies on this aspect must be performed using surrogates and/or human noroviruses. On the other hand, the opportunities are such that there is still much room for improvements from the proof-of-concept results in this study if the antiviral activity is confirmed using norovirus surrogates and human noroviruses. There is also opportunity to develop approaches for effective incorporation/utilization of these NPs in practical antiviral agents would be needed. Potential applications of such NPs-incorporated antiviral agents would be disinfectants/sanitizers for decontaminating norovirus contaminated surfaces, especially in hospital or clinical settings, since contaminated surfaces have been reported as a secondary outbreak in many reported norovirus outbreaks because of inadequate disinfection [54, 71]. Rapid and effective antiviral action of NPs-based disinfectants would be useful in preventing the spread of infection by reducing secondary transfer from contaminated surfaces in these settings.

## Conclusions

The current lack of rapid, point-of-care inactivation strategies hinders progress in controlling frequent and widespread norovirus outbreaks. Because the most promising virucides directly bind to the viral capsid [43], inactivation via nanoparticles (NPs) is a more universal alternative to other physical and chemical strategies that exhibit variable efficacy. Using GI.1 VLPs as a model viral system, this study provides proof of concept that Au/CuS core/shell NPs can rapidly inactivate human norovirus. Immunoblotting, dynamic light scattering, and TEM results provided evidence that capsid protein degradation and capsid damage appeared to be the mechanisms associated with inactivation and have a direct dependence on both NPs concentration and treatment time. Nevertheless, this study demonstrated that Au/CuS NPs are promising antivirals, although further studies using HuNoV-rich fecal extracts will be beneficial to confirming the efficacy of Au/CuS NPs against norovirus.

## Supporting Information

S1 Fig Mean size of capsids and/or nanoparticles. (DOCX)
