# Supplementary material for: Antiviral Activity of Gold/Copper Sulfide Core/Shell Nanoparticles against Human Norovirus Virus-Like Particles
Source: PLoS One. 2015 Oct 16;10(10):e0141050. doi: 10.1371/journal.pone.0141050 (PMC4608711; doi:10.1371/journal.pone.0141050)
Supplement: S1 Fig — (DOCX) [file pone.0141050.s001.docx]

# Supporting Information

**A**

**B**

**S1 Fig. Mean size of capsids and/or nanoparticles.** Particle sizes in VLP, NP, and VLP + NP solutions after a 10-min treatment with (A) 0.83 or (B) 1.245 µM Au/CuS nanoparticles. All solutions contain an equivalent volume of VLPs and/or NPs, and all were rotated end-over-end and centrifuged for 10 and 5 min, respectively. Particle size was measured using dynamic light scattering.
